# Risk of COVID-19 and its complications in patients with atopic dermatitis undergoing dupilumab treatment—a population-based cohort study

**DOI:** 10.1007/s12026-021-09234-z

**Published:** 2021-10-13

**Authors:** Khalaf Kridin, Yochai Schonmann, Arie Solomon, Erez Onn, Dana Tzur Bitan, Orly Weinstein, Arnon D. Cohen

**Affiliations:** 1 Lübeck Institute of Experimental Dermatology, University of Lübeck, Ratzeburger Allee 160, 23562 Lübeck, Germany; 2 Azrieli Faculty of Medicine, Bar-Ilan University, Safed, Israel; 3 Unit of Dermatology and Skin Research Laboratory, Baruch Padeh Medical Center, Poriya, Israel; 4 Clalit Health Services, Tel-Aviv, Israel; 5 Baruch Padeh Medical Center, Poriya, Tiberias, Israel; 6 Department of Behavioral Sciences, Ariel University, Ariel, Israel; 7 Faculty of Health Sciences, Ben-Gurion University of the Negev, Ben-Gurion Ave, Beer Sheva, Israel

**Keywords:** AD, Atopic dermatitis, COVID-19, Biologics

## Abstract

The risk of coronavirus disease (COVID-19) infection and its complications among patients with atopic dermatitis (AD) treated by dupilumab is yet to be determined. We aimed to assess the risk of SARS-CoV-2 infection, COVID-19-associated hospitalization, and mortality among patients with AD treated by dupilumab. A population-based cohort study was conducted to compare AD patients treated by dupilumab (*n* = 238) with those treated by prolonged systemic corticosteroids (≥ 3 months; *n* = 1,023), phototherapy (*n* = 461), and azathioprine or mycophenolate mofetil (MMF; *n* = 194) regarding the incidence of COVID-19 and its complications. The incidence rate of COVID-19, COVID-19-associated hospitalization, and mortality among patients treated by dupilumab was 70.1 (95% CI, 40.5–116.4), 5.0 (95% CI, 0.3–24.7), and 0.0 per 1,000 person-year, respectively. The use of dupilumab was not associated with an increased risk of SARS-CoV-2 infection [adjusted HR for dupilumab vs. prolonged systemic corticosteroids: 1.13 (95% CI, 0.61–2.09); dupilumab vs. phototherapy: 0.80 (95% CI, 0.42–1.53); dupilumab vs. azathioprine/MMF: 1.10 (95% CI, 0.45–2.65)]. Dupilumab was associated with a comparable risk of COVID-19-associated hospitalization [adjusted HR for dupilumab vs. prolonged systemic corticosteroids: 0.35 (95% CI, 0.05–2.71); dupilumab vs. phototherapy: 0.43 (95% CI, 0.05–3.98); dupilumab vs. azathioprine/MMF: 0.25 (95% CI, 0.02–2.74)]. When applicable, the risk of mortality was not elevated in patients with AD treated by dupilumab [HR for dupilumab vs. prolonged systemic corticosteroids: 0.04 (95% CI, 0.00–225.20)]. To conclude, dupilumab does not impose an increased risk of SARS-CoV-2 infection or COVID-19 complications in patients with AD. Dupilumab should be continued and considered as a safe drug for moderate-to-severe AD during the pandemic.

## Introduction

Dupilumab is a novel anti-interleukin-4 (IL-4) receptor-α monoclonal antibody that targets the signaling pathways of IL-4 and IL-13, which are key type 2 cytokines in the pathophysiology of atopic dermatitis (AD). Randomized clinical trials have demonstrated that adults with moderate-to-severe AD managed by weekly or biweekly dupilumab injections experienced significantly improved clinical and patient-reported outcomes [1, 2]. While dupilumab was not implicated with an increased risk of infections [3], the relative safety of this emerging drug during the ongoing coronavirus disease 2019 (COVID-19) pandemic, relative to other systemic therapeutic modalities, is yet to be estimated.

Given that severe COVID-19 is typified by a hyperinflammatory state which mediates the late complications of the disease [4, 5], it is of great interest to investigate whether exposure to immunomodulatory drugs influences the manifestations of the disease. Inconsistent findings have accumulated with regard to the role exerted by immunomodulatory drugs on the clinical phenotype and prognostic outcomes of SARS-CoV-2 infection [6–11]. In the current study, we sought to investigate the risk of COVID-19, COVID-19-associated hospitalization, and mortality among patients with AD treated by dupilumab. To precisely evaluate the safety of this treatment during the pandemic, patients with AD on dupilumab were compared with three reference groups: (i) patients with AD treated by long-term systemic corticosteroids, (ii) phototherapy, and (iii) azathioprine or mycophenolate mofetil (MMF).

## Methods

### Study design and dataset

We carried out a retrospective cohort study that tracked patients with AD to assess the incidence of COVID-19 infection, COVID-associated hospitalization, and mortality under different treatment regimens.

The current study took advantage of the computerized database of Clalit Health Services (CHS). CHS is the largest insurer in Israel, covering a wide variety of private and public healthcare services for 4,540,768 enrollees as of October 2018. CHS database retrieves data from numerous sources originating both from ambulatory and hospitalized care settings. CHS is additionally typified by a minor loss to follow-up and free access to healthcare services. All these characteristics render the CHS database highly compatible with the production of reliable and valid epidemiological data [12]. The study was approved by the institutional review board (IRB) in accordance with the Declaration of Helsinki (approval code: 0212–17-COM).

### Study population and definition of exposure

The computerized database of CHS was systematically screened for all prevalent cases with a diagnostic code of AD as registered by board-certified dermatologists. Eligible patients had to be alive and older than 18 years of age at the onset of the pandemic, defined as the date of the first confirmed case of COVID-19 in Israel (February 27, 2020).

Patients were considered to be exposed to an AD-related when there were two prescriptions extending on more than 1 month. Patients with AD receiving dupilumab were compared to three different reference groups of patients with AD treated by (i) systemic corticosteroids for more than 3 months, (ii) phototherapeutic modalities (ultraviolet B [UVB], psoralen and ultraviolet A [PUVA]), and (iii) azathioprine or MMF.

Exposure to systemic corticosteroids was defined only if the drug preceded the diagnosis of COVID-19 by at least 1 month (to exclude cases in which systemic corticosteroids were given to treat symptoms of advanced COVID-19 infection). Azathioprine and MMF were pooled into a single reference group given that both drugs share a similar mechanism of action inhibiting the purine pathway and cell proliferation [13], and that the groups of patients managed by these two agents are homogenous. Cyclosporine and methotrexate were not assigned as referent groups due to the small number of patients treated with these drugs (92 and one patients, respectively).

Patients managed by dupilumab, long-term systemic corticosteroids, phototherapy, or azathioprine/MMF in conjunction with other concomitant systemic immunomodulatory or immunosuppressive drugs were excluded from the analysis in order to evaluate the independent influence of the drug of interest. Concomitant drugs warranting exclusion were cyclosporine, methotrexate, and omalizumab.

### Definition of COVID-19-related outcomes

The medical records of eligible patients were screened for documentation of COVID-19. The diagnosis of COVID-19 was established upon US FDA-approved molecular tests. COVID-19-associated hospitalization was defined in COVID-19-confirmed patients admitted to intensive care units, internal medicine, or COVID-19-specific respiratory inpatient wards. COVID-19-associated mortality was defined in COVID-19-confirmed patients whose cause of death was ascribed to COVID-19 or its complications.

Study participants’ date of death was ascertained by linking the study cohort with the Ministry of Interior registry. All study participants were followed up from the onset of the pandemic in Israel or the date of drug initiation, whichever occurs later, until January 6, 2021, drug discontinuation, death, or fulfilling the study outcomes, whichever occurs earlier.

### Covariates

Outcome measures were adjusted for the following comorbidities: chronic obstructive pulmonary disease (COPD), ischemic heart disease (IHD), diabetes mellitus, chronic renal failure (CRF), hypertension, hyperlipidemia, and malignancies, all of which were found to predict worse prognostic outcomes of COVID-19 [14–18]. COVID-19 outcomes were additionally controlled for smoking owing to the association of the latter with inferior outcomes of COVID-19 [14, 15]. The CHS registry for chronic diseases was utilized to identify comorbidities of eligible patients prior to the development of COVID-19.

### Statistical analysis

Incidence rates of outcomes were calculated and expressed as the number of events per 1,000 person-years. Hazard ratios (HRs) for the risk of incident outcomes were obtained by the use of Cox regression model. Two-tailed *p* values less than 0.05 were considered statistically significant. All statistical analyses were performed using SPSS software, version 25 (SPSS, Armonk, NY: IBM Corp).

## Results

### Characteristics of the study population

Out of 78,073 adult patients with AD, four treatment groups were identified and included in the study. Of all, 238, 1,023, 461, and 194 patients with AD were managed by dupilumab, long-term systemic corticosteroids, phototherapy, and azathioprine/MMF during the pandemic, respectively. Table 1 delineates the demographic and clinical characteristics of study participants. Compared to those treated by systemic corticosteroids during the pandemic, patients under dupilumab were younger, had lower BMI, and lower prevalence of preexisting comorbidities (Table 1).

**Table 1:** Descriptive characteristics of the study population

Characteristic	Dupilumab (*N* = 238)	Systemic corticosteroids ≥ 3 months (*N* = 1,023)	Phototherapy (*N* = 461)	Azathioprine and mycophenolate mofetil (*N* = 194)
Age at the onset of pandemic, years				
Mean (SD)	49.2 (19.9)	59.5 (19.7)	49.9 (20.8)	49.4 (19.8)
Age at the onset of the disease, years				
Mean (SD)	41.4 (22.2)	52.1 (20.5)	44.8 (22.3)	41.5 (20.4)
BMI, kg/m^2^				
Mean (SD)	26.0 (5.1)	27.3 (5.9)	26.1 (5.1)	26.5 (6.0)
Sex, *n* (%)				
Male	141 (59.2%)	436 (42.6%)	217 (47.1%)	84 (43.3%)
Female	97 (40.8%)	587 (57.4%)	244 (52.9%)	110 (56.7%)
Ethnicity, *n* (%)				
Jews	209 (87.8%)	855 (83.6%)	403 (87.4%)	166 (85.6%)
Arabs	29 (12.2%)	168 (16.4%)	58 (12.6%)	28 (14.4%)
Smoking, *n* (%)	94 (39.5%)	397 (38.8%)	163 (35.4%)	62 (32.0%)
COPD, *n* (%)	17 (7.1%)	162 (15.8%)	22 (4.8%)	15 (7.7%)
Diabetes mellitus, *n* (%)	46 (19.3%)	285 (27.9%)	70 (15.2%)	43 (22.2%)
Hypertension, *n* (%)	63 (26.5%)	428 (41.8%)	106 (23.0%)	82 (42.3%)
Hyperlipidemia, *n* (%)	100 (42.0%)	642 (62.8%)	190 (41.2%)	103 (53.1%)
Ischemic heart disease, *n* (%)	24 (10.1%)	177 (17.3%)	50 (10.8%)	29 (14.9%)
Malignancy, *n* (%)	33 (13.9%)	273 (26.7%)	67 (14.5%)	33 (17.0%)
Chronic renal failure, *n* (%)	10 (4.2%)	135 (13.2%)	20 (4.3%)	49 (25.3%)

Abbreviations: *n*, number; *SD*, standard deviation; *BMI*, body mass index

### Primary analysis comparing the risk of COVD-19 outcomes associated with dupilumab relative to systemic corticosteroids

The incidence rate of COVID-19, COVID-19-associated hospitalization, and COVID-19-associated mortality in the dupilumab group was estimated at 70.1 (95% CI, 40.5–116.4), 5.0 (95% CI, 0.3–24.7), and 0.0 per 1,000 person-years, respectively. The corresponding incidence rates in the systemic corticosteroids group were 63.6 (95% CI, 48.1–82.5), 23.7 (95% CI, 14.9–36.0), and 7.1 (95% CI, 2.9–14.7) per 1,000 person-years, respectively (Table 2).

**Table 2:** The risk of COVID-19 and its complications among patients with atopic dermatitis treated by dupilumab compared to those treated by systemic corticosteroids for ≥ 3 months during the pandemic

	COVID-19 infection		COVID-19-associated hospitalization		COVID-19-associated mortality	
	Dupilumab (*N* = 238)	Systemic corticosteroids ≥ 3 months (*N* = 1,023)	Dupilumab (*N* = 238)	Systemic corticosteroids ≥ 3 months (*N* = 1,023)	Dupilumab (*N* = 238)	Systemic corticosteroids ≥ 3 months (*N* = 1,023)
Follow-up time, PY	197.0	833.9	199.8	843.3	200.1	847.2
Median follow-up time, months (range)	10.3 (4.3–10.3)	10.3 (0.3–10.3)	10.3 (4.3–10.3)	10.3 (0.3–10.3)	10.3 (4.3–10.3)	10.3 (0.3–10.3)
Number of events	14	53	1	20	0	6
Incidence rate/1000 PY (95% CI)	70.1 (40.5–116.4)	63.6 (48.1–82.5)	5.0 (0.3–24.7)	23.7 (14.9–36.0)	0.0	7.1 (2.9–14.7)
Unadjusted HR (95% CI) [*p* value]	1.11 (0.62–2.01) [0.720]	Reference	0.21 (0.03–1.56) [0.127]	Reference	0.04 (0.00–225.20) [0.456]	Reference
Age- and sex-adjusted HR (95% CI) [*p* value]	1.10 (0.60–2.02) [0.755]	Reference	0.28 (0.04–2.13) [0.220]	Reference	NA [0.984]	Reference
Fully adjusted HR (95% CI) [*p* value]^a^	1.13 (0.61–2.09) [0.699]	Reference	0.35 (0.05–2.71) [0.317]	Reference	NA [0.975]	Reference

^a^Multivariate logistic regression model adjusting for age, sex, COPD, CRF, IHD, HTN, hyperlipidemia, obesity, malignancy, diabetes mellitus, and smoking

Abbreviations: *n*, number; *PY*, person-year; *HR*, hazard ratio; *CI*, confidence interval; *NA*, non-applicable

Bold: significant value

The risk of COVID-19 (fully adjusted HR, 1.13; 95% CI, 0.61–2.09; *p* = 0.699), COVID-19-asscoaited hospitalization (fully adjusted HR, 0.35; 95% CI, 0.05–2.71; *p* = 0.317), and COVID-19-asscoaited mortality (HR, 0.04; 95% CI, 0.0–225.2; *p* = 0.456) was not statistically different between patients treated by dupilumab and systemic corticosteroids (Table 2).

### Primary analysis comparing the risk of COVD-19 outcomes associated with dupilumab relative to phototherapy and azathioprine/MMF

In patents with AD under phototherapy during the pandemic, the incidence rate of COVID-19 infection and COVID-19-asscoaited hospitalization was 79.5 (95% CI, 54.6–112.0) and 10.4 (95% CI, 3.3–25.0) per 1,000 person-years, respectively. Relative to patients with AD managed by phototherapy, those managed by dupilumab displayed comparable COVID-19 infection (fully adjusted HR, 0.80; 95% CI, 0.42–1.53; *p* = 0.500) and COVID-19-associated hospitalization (fully adjusted HR, 0.43; 95% CI, 0.05–3.98; *p* = 0.458; Table 3).

**Table 3:** The risk of COVID-19 and its complications among patients with atopic dermatitis treated by dupilumab compared to those treated by phototherapy during the pandemic

	COVID-19 infection		COVID-19-associated hospitalization		COVID-19-associated mortality	
	Dupilumab (*N* = 238)	Phototherapy (*N* = 461)	Dupilumab (*N* = 238)	Phototherapy (*N* = 461)	Dupilumab (*N* = 238)	Phototherapy (*N* = 461)
Follow-up time, PY	197.0	377.4	199.8	386.1	200.1	387.8
Median follow-up time, months (range)	10.3 (4.3–10.3)	10.3 (1.0–10.3)	10.3 (4.3–10.3)	10.3 (1.3–10.3)	10.3 (4.3–10.3)	10.3 (5.9–10.3)
Number of events	14	30	1	4	0	0
Incidence rate/1000 PY (95% CI)	70.1 (40.5–116.4)	79.5 (54.6–112.0)	5.0 (0.3–24.7)	10.4 (3.3–25.0)	0.0	0.0
Unadjusted HR (95% CI) [*p* value]	0.89 (0.47–1.68) [0.723]	Reference	0.48 (0.05–4.33) [0.516]	Reference	NA	Reference
Age- and sex-adjusted HR (95% CI) [*p* value]	0.81 (0.43–1.54) [0.528]	Reference	0.44 (0.05–3.96) [0.462]	Reference	NA	Reference
Fully adjusted HR (95% CI) [*p* value]^a^	0.80 (0.42–1.53) [0.500]	Reference	0.43 (0.05–3.98) [0.458]	Reference	NA	Reference

^a^Multivariate logistic regression model adjusting for age, sex, COPD, CRF, IHD, HTN, hyperlipidemia, obesity, malignancy, diabetes mellitus, and smoking

Abbreviations: *n*, number; *PY*, person-year; *HR*, hazard ratio; *CI*, confidence interval; *NA*, non-applicable

Bold: significant value

The incidence rate of COVID-19 infection and COVID-19-associated hospitalization in the azathioprine/MMF group was 55.9 (95% CI, 27.3–102.6) and 18.5 (95% CI, 4.7–50.3), respectively. When compared to patients with AD treated by azathioprine/MMF, patients under dupilumab did not demonstrate statistically different COVID-19 infection (fully adjusted HR, 1.10; 95% CI, 0.45–2.65; *p* = 0.840) or COVID-19-associated hospitalization (fully adjusted HR, 0.25; 95% CI, 0.02–2.74; *p* = 0.254; Table 4).

**Table 4:** The risk of COVID-19 and its complications among patients with atopic dermatitis treated by dupilumab compared to those treated by azathioprine and mycophenolate mofetil during the pandemic

	COVID-19 infection		COVID-19-associated hospitalization		COVID-19-associated mortality	
	Dupilumab (*N* = 238)	Azathioprine and MMF (*N* = 194)	Dupilumab (*N* = 238)	Azathioprine and MMF (*N* = 194)	Dupilumab (*N* = 238)	Azathioprine and MMF (*N* = 194)
Follow-up time, PY	197.0	161.0	199.8	162.3	200.1	163.5
Median follow-up time, months (range)	10.3 (4.3–10.3)	10.3 (3.6–10.3)	10.3 (4.3–10.3)	10.3 (3.8–10.3)	10.3 (4.3–10.3)	10.3 (7.8–10.3)
Number of events	14	9	1	3	0	0
Incidence rate/1000 PY (95% CI)	70.1 (40.5–116.4)	55.9 (27.3–102.6)	5.0 (0.3–24.7)	18.5 (4.7–50.3)	0.0	0.0
Unadjusted HR (95% CI) [*p* value]	1.27 (0.55–2.94) [0.572]	Reference	0.27 (0.03–2.61) [0.258]	Reference	NA	Reference
Age- and sex-adjusted HR (95% CI) [*p* value]	1.24 (0.53–2.89) [0.622]	Reference	0.32 (0.03–3.11) [0.324]	Reference	NA	Reference
Fully adjusted HR (95% CI) [*p* value]^a^	1.10 (0.45–2.65) [0.840]	Reference	0.25 (0.02–2.74) [0.254]	Reference	NA	Reference

^a^Multivariate logistic regression model adjusting for age, sex, COPD, CRF, IHD, HTN, hyperlipidemia, obesity, malignancy, diabetes mellitus, and smoking

Abbreviations: *n*, number; *PY*, person-year; *MMF*; mycophenolate mofetil; *HR*, hazard ratio; *CI*, confidence interval; *NA*, non-applicable

Bold: significant value

No events of COVID-19-associated mortality occurred in the dupilumab, phototherapy, and azathioprine/MMF groups. Therefore, HRs for this outcome could not be calculated (Tables 3 and 4).

## Discussion

The current retrospective large-scale study provides the first population-based estimate of the outcomes of COVID-19 among patients treated with dupilumab during the pandemic. Patients with AD treated by dupilumab exhibited no increased risk of SARS-CoV-2 infection or COVID-19-associated hospitalization and mortality. The latter conclusions were drawn following a comparison with three different reference groups consisting of patients with AD on long-term systemic corticosteroids, phototherapy, and azathioprine/MMF.

### The current literature

While dupilumab is a relatively new drug approved only in 2017, a compelling body of evidence accumulated to suggest its highly favorable safety profile. In three randomized, placebo-controlled phase III clinical trials (SOLO 1, SOLO 2, and CHRONOS), the incidence rate of infection was comparable between dupilumab-treated patients and placebo-controlled patients [1, 2]. Nasopharyngitis was the most frequently encountered infection in these clinical trials [1–3].

The occurrence of SARS-CoV-2 infection among patients managed by dupilumab has been anecdotally reported in patients with AD [19–22], asthma [23–25], and chronic rhinosinusitis [26]. The majority of these patients were reported to follow a mild course of COVID-19, with only three patients manifesting with mild-to-severe symptoms necessitating admission [20, 22, 24] and none expiring due to the infection. However, these absolute numbers lack meaningfulness unless validated by controlled observational studies that enroll comparator groups and investigate the influence exerted by dupilumab on the outcomes of COVID-19 relative to other AD-related drugs.

Three retrospective studies evaluated COVID-19 outcomes among hospital-based cohorts of patents managed by dupilumab in Italy, a country severely hit by the pandemic. Using telephone interviews, 200 patients with AD under dupilumab were followed between March 11, 2020 and April 22, 2020 in Naples. None of these eligible patients were tested positive for COVID-19 [27]. In a study of similar methodology performed in Brescia, 71 dupilumab-treated AD patients have been followed. Of whom, two (2.8%) developed SARS-CoV-2 infection, and one (1.4%) was hospitalized [20]. In a cohort of 245 patients in Milan, two (0.8%) patients acquired the infection, of whom one (0.4%) patient was admitted to an inpatient ward [22]. Using a retrospective analysis of a large international COVID-19 cohort, patients under dupilumab, for all indications, were found to exhibit lower rates of mechanical ventilation and mortality [28].

### Implications and interpretation of the study findings

Utilizing a large-scale population-based study, we could attest that dupilumab does not place patients with AD at an increased risk of worse outcomes of COVID-19. The incidence rate of COVID-19-associated hospitalization, an outcome measure that accurately mirrors the severity of SARS-CoV-2 infection, was numerically lower among dupilumab-treated AD patients relative to the three comparators. However, this difference fell short of statistical significance. It is conceivable to assume that employing a larger sample size and longer follow-up duration may have rendered the study more statistically powered to disclose significant differences. Further studies with the aforementioned features are, therefore, warranted to better delineate the safety of dupilumab during the pandemic.

Our findings align with the declaration announced by the European Academy of Dermatology and Venereology (EADV) task force for AD, in which dupilumab was not considered to increase the risk for viral infections and might thus be preferred over conventional systemic immunosuppressive drugs during the pandemic [29]. However, this hypothetical advantage was not corroborated by robust clinical data, thus leaving the medical literature in a desperate need to validate this approach. Our study, alongside other lines of evidence and expert opinions, argues in favor of continuing the drug during the pandemic. In addition, dupilumab should be positively considered in patients with severe AD, necessitating the administration of systemic drugs.

The current understanding of the pathomechanism of COVID-19 suggests that angiotensin enzyme 2 (ACE2) is the host functional receptor for SARS-CoV-2, which hyperactivates an immune response predominated by T-helper (Th)-1 cells which results in lung impairment [30]. Given that IL-4 and IL-13 targeted by dupilumab are Th-2 cytokines, their signaling pathways have not been classically implicated in the cytokine storm typifying severe advanced COVID-19 [27, 31]. However, recent studies demonstrated that the levels of IL-13 were significantly higher among COVID-19-positive patients as compared to their uninfected counterparts [28, 32–34], as well as among COVID-19 patients requiring mechanical ventilation relative to milder COVID-19 patients [28]. In SARS-CoV-2 infected mice, IL-13 inhibition reduced death and disease severity without affecting viral load, denoting an immunopathogenic role for this cytokine in mediating the hyperinflammatory state in COVID-19 [28]. These experimental findings lend weight to our epidemiological observation indicating a non-elevated risk of COVID-19-associated hospitalization and mortality under an IL-13 blocking agent, dupilumab.

### Strengths and limitations

The current study provides novel data regarding the risk of COVID-19 outcomes under dupilumab. Recruitment of three unrelated reference groups enables to evaluate the relative risk of COVID-19 outcomes in patients with AD under dupilumab as compared to AD patients treated with other agents. Since the CHS dataset enables comprehensive access to the whole spectrum of healthcare services and owing to the notification requirement, COVID-19 cases could not be overlooked, even those occurring years following the initial diagnosis of AD. The latter feature overcomes the substantial loss to follow that frequently interferes with the validity of hospital-based studies. The study was undertaken in a country with a high incidence of COVID-19, thus allowing the detection and profiling of COVID-19-positive AD patients. Limitations to be acknowledged pertain to the small number of outcome events (COVID-19-associated hospitalization and mortality), the relatively short duration of follow-up, and lack of data regarding severity measurements of AD.

In conclusion, patients with AD managed by dupilumab do not experience an increased risk of SARS-CoV-2 infection, COVID-19-hospitalization, or mortality when compared to patients with AD managed by systemic corticosteroids, phototherapy, and azathioprine/MMF. These findings substantiate the approach suggesting avoiding preventive cessation of dupilumab unless indicated individually by the patient’s clinical data, comorbidities, or specific risk factors. In moderate-to-severe AD necessitating systemic treatment, dupilumab should be positively considered as a safe therapeutic modality during the pandemic. This drug should be continued in those who were already placed on it before the pandemic. Additional studies with longer follow-up durations are necessary to better delineate the influence of this drug on COVID-19.

## Data Availability

The datasets generated and/or analyzed during the current study are available from the corresponding author on reasonable request.
